# What Basophil Testing Tells Us About CSU Patients – Results of the CORSA Study

**DOI:** 10.3389/fimmu.2021.742470

**Published:** 2021-09-28

**Authors:** João Marcelino, Katrine Baumann, Per Stahl Skov, Maria Conceição Pereira Santos, Inga Wyroslak, Jörg Scheffel, Sabine Altrichter, Anders Woetmann, Manuel Pereira-Barbosa, Célia Costa, Marcus Maurer

**Affiliations:** ^1^ Immunoallergology Department, Hospital Santa Maria, Centro Hospitalar Universitário Lisboa Norte E.P.E., Lisbon, Portugal; ^2^ Dermatological Allergology, Allergie-Centrum-Charité, Department of Dermatology and Allergy, Charité – Universitätsmedizin Berlin, Berlin, Germany; ^3^ Fraunhofer Institute for Translational Medicine and Pharmacology ITMP, Allergology and Immunology, Berlin, Germany; ^4^ Contract Research Dprt., RefLab ApS, Copenhagen, Denmark; ^5^ Odense Research Center for Anaphylaxis (ORCA), Urticaria Center of Reference and Excellence (UCARE), Odense University Hospital, Odense, Denmark; ^6^ Laboratory of Clinical Immunology, Faculdade de Medicina, Instituto de Medicina Molecular, Universidade de Lisboa, Lisbon, Portugal

**Keywords:** angioedema, autologous serum skin test (ASST), basophil activation test (BAT), basophil histamine release test, chronic spontaneous urticaria (CSU), anti-thyroperoxidase (anti-TPO), IgE (immunoglobulin E)

## Abstract

Basophil testing is the most effective single approach for diagnosing type-IIb autoimmune chronic spontaneous urticaria (TIIbaiCSU). A positive basophil test has been linked to long disease duration, higher disease activity, a poor response to antihistamines and omalizumab, and a better response to cyclosporine and fenebrutinib. As of now it is unclear what other features are connected to a positive basophil test in chronic spontaneous urticaria (CSU). We aimed to identify features of basophil test-positive CSU patients. We performed a cross-sectional study of 85 CSU patients. Basophil testing was done with the basophil activation test (BAT) and the basophil histamine release assay (BHRA). Data were analysed using SPSS: Student’s t-test, Chi-square test, Odds Ratio, Spearman’s correlation test. Of 85 CSU patients, 44% and 28% tested positive with the BAT and BHRA, respectively. These patients showed higher disease activity and impact, lower levels of disease control and total serum IgE, as well as higher rates of having a positive autologous serum skin test (ASST), angioedema, nocturnal symptoms, symptoms for >5 days/week, and thyroid autoantibodies. The ASST, by itself, was not a good predictor of basophil test results, but it predicted a positive basophil test in up to 100% of cases when combined with angioedema, thyroid autoantibodies or low IgE. In conclusion, a positive basophil test is linked to known features of TIIbaiCSU and novel characteristics including nocturnal symptoms. Further studies on basophil test-positive and -negative CSU patients can help to better understand CSU endotypes and to develop better management approaches.

## Introduction

Chronic spontaneous urticaria (CSU) is a common and debilitating disease, affecting adults and children, with a marked impact on patients’ quality of life (1–5). It is defined by the spontaneous appearance of transient itchy wheals (hives), angioedema, or both for more than 6 weeks (1–6). Recent advances have characterized CSU as an autoantibody-driven disease, where mast cells and basophils in the skin are activated through two distinct pathways (4, 5).

In type I autoimmune CSU (TIaiCSU, also called autoallergic CSU), IgE autoantibodies are cross-linked by self-antigens, for example thyroid peroxidase or interleukin 24 (2–5). In type IIb autoimmune CSU (TIIbaiCSU), IgG and IgM autoantibodies are directed against IgE receptors (or IgE itself) on the surface of mast cells and basophils (1–7).

In clinical practice, it is important to know if a patient has TIIbaiCSU, as TIaiCSU and TIIbaiCSU present distinct phenotypes. Patients with TIIbaiCSU have been shown to have an increased risk of developing other autoimmune diseases, of failing antihistamine and omalizumab treatment, and of having a better response to cyclosporine and fenebrutinib (8–11). They have also been linked to higher disease activity and impact, making effective treatment both critical and challenging. The identification of further characteristics that characterize both subgroups is still a matter of ongoing research (4).

TIIbaiCSU is diagnosed by the combination of three tests, i.e. the autologous serum skin test (ASST), the basophil activation test (BAT) or the basophil histamine release assay (BHRA), and an ELISA or Western Blot-based autoantibody assay (1). Testing of all CSU patients for TIIBaiCSU with these three test is difficult, for several reasons, including their availability and the high and increasing prevalence of CSU, of up to 1.4% (12).

Basophil testing, with the BAT or the BHRA, is the single best diagnostic test for TIIbaiCSU. The recent PURIST study found that the BAT and the BHRA were 69% and 88% predictive of TIIbaiCSU, respectively (4). In addition, several reports show that the BAT/BHRA alone can identify patients with higher disease activity, longer disease duration, and poorer response to omalizumab (1, 9, 13, 14). In contrast, the ASST, initially thought off as a good, inexpensive, globally available marker of TIIbaiCSU, is only 27% predictive of TIIbaiCSU (4). Moreover, the ASST is influenced by the intake of antihistamines (the first-line treatment and a treatment difficult to interrupt in patients with severe urticaria), it does not give a quantifiable result, and handling of biologic samples requires exceptional care, as reinjecting patients’ sera has important safety requirements (15).

Basophil testing of patients with CSU is performed at many urticaria centers of reference and excellence [UCAREs (16)] and by a limited number of commercial vendors. Finding ways of selecting patients who benefit the most from performing TIIbaiCSU tests are needed; as diagnosing CSU patients with TIIbaiCSU has real implications for the patient and the physician.

The present CORSA (component-resolved screening for autoimmune chronic spontaneous urticaria) study has two main objectives: 1) to confirm known and identify as of yet unknown features of BAT/BHRA-positive CSU patients, 2) to identify combinations of clinical and laboratory markers of CSU patients that can help to guide patient selection for basophil testing.

## Materials and Methods

### Patient Selection and Study Conduct

We performed a cross-sectional, single-centre study of 85 patients with CSU with daily symptoms. Patients were evaluated during a 1-year follow-up to evaluate the therapy needed to achieve CSU control. Patients were considered eligible if they had active CSU, and if they were not, nor had ever been, on omalizumab or cyclosporine. Patients were excluded if they had not performed all 3 of the following: the ASST, the BAT and the BHRA.

The CORSA study was approved by the corresponding ethics committee: *Comissão de Ética do Centro Hospitalar Universitário Lisboa Norte e Centro Académico Médico de Lisboa* (Ethics Committee authorization references 129/17 and 339/19). The study was conducted according to the Declaration of Helsinki, Good Clinical Practice and local regulations. All patients provided written informed consent.

### Clinical Assessment of Patients

Data collected included: 1) date of CSU onset, 2) CSU duration, 3) presence of angioedema, 4) hives duration, 5) number of symptomatic days per week, 6) therapy necessary to achieve control (evaluated 12 months after patient enrolment), 7) presence of comorbid autoimmune diseases, 8) CSU activity as assessed with the Urticaria Activity Score 7 [UAS7 (9, 17)] CSU impact, evaluated with the Dermatology Life Quality Index [DLQI (18, 19)], and (8) CSU control per the Urticaria Control Test [UCT (20)]. The DLQI and UCT questionnaires were filled on the day of the ASST, and the UAS7 was calculated for the week prior to the ASST.

The ASST and BAT were performed according to clinical practice, and not for the purposes of the study. The BHRA was performed for this study from surplus serum collected from the patients.

### Additional Measurements

Additional assessments included patient age, gender, total serum IgE, C3, C4, CH50, thyroid-stimulating hormone, free T4, thyroid auto-antibodies (anti-Tg and anti-TPO), ANA, anti-dsDNA, complete blood count, C-reactive protein, erythrocyte sedimentation rate, protein electrophoresis, immunoglobulins, liver and kidney function.

Patient history information was collected on a first visit to our urticaria outpatient clinic at our Immunoallergology Department. A second visit was scheduled within one month of the first visit. Patients were instructed to stop any antihistamine medication for 5 days and any systemic corticosteroids for at least 2 weeks prior to their second visit. On their second visit, venous blood was drawn to perform the ASST. The same blood was used to perform the BAT, the BHRA and the remaining blood tests. The UAS7, DLQI and UCT were collected on their second visit as well.

### The Autologous Serum Skin Test (ASST)

The ASST was performed as previously reported (15, 21, 22). Venous blood was collected on the day of the test, centrifuged and the serum was used to perform the ASST (the same serum was also used to perform the BAT and BHRA). An intradermal test was performed, by injecting 50 µl of serum with an insulin needle and reading after 30 minutes. The test was considered positive when the diameter of the serum induced wheal was ≥1.5mm larger than that induced by saline and erythema was present.

### The Basophil Activation Test (BAT)

The BAT was carried out by the Laboratory of Clinical Immunology, Faculdade de Medicina, Instituto de Medicina Molecular, Universidade de Lisboa, Lisbon, Portugal. The serum from the patients was exposed to heat complement-inactivation, for 30 minutes, before being tested at dilutions of 1:1, 1:5, 1:10. A positive control with N-formyl-L-methionyl-L-leucyl-phenylalanine (fMLP) and anti-FcϵRI, and a negative control with saline solution (NaCl 0,9%), were used. IL-3 (stimulation buffer) and donor basophils were added to each tube, and double staining was performed with anti-CCR3-PE and anti-CD63-FITC monoclonal antibodies (Bühlmann, Switzerland) and incubated at 37°C for 15 minutes. Afterwards, erythrocytes were lysed for 10 minutes, and then the samples were washed (PBS buffer) and resuspended with the same solution. Data acquisition was performed by flow cytometry FACSCalibur (Becton-Dickinson Immunocytometry System, CA). The basophil population was identified as CCR3+ cells, and basophil activation was expressed as a proportion of CD63+ basophils corrected for the negative control and as a ratio of CD63 (%) of activated cells and negative control – stimulation index (SI). Data analysis was performed by Flow Jo (TreeStar, Ashland, or USA). Each serum was tested with basophils from three donors and the results were considered positive when there was a basophil activation percentage ≥ 5% activation, in response to the 1:1 serum dilution, and a stimulation index (SI) ≥2 in the basophils of at least one donor.

### The Basophil Histamine Release Assay (BHRA)

The BHRA was carried out by RefLab ApS, Copenhagen, Denmark. Four buffy coats (obtained from the Danish National Hospital; Rigshospitalet, Copenhagen, Denmark), containing healthy donor basophils, were stored at 2-8°C overnight with IL-3 in a final concentration of 1 ng/ml. Buffy coats were washed in saline the following day and surface IgE was partially removed using a stripping buffer (pH 3.6, RefLab, Copenhagen, Denmark) before the cells were resuspended in Pipes buffer (RefLab, Copenhagen, Denmark). Stripped buffy coats were incubated at 37°C for 60 min with 20% patient serum and supernatants were then extracted for histamine quantification in the histamine release (HR) assay: Glass fiber-coated microtiter plates were loaded with 25 µl of each supernatant and incubated for 1 hour before histamine was measured using the ortho-phthaldialdehyde method and a highly sensitive fluorometer (Histareader 501, RefLab, Copenhagen, Denmark) according to RefLab instructions. The total histamine content was determined by lysing the basophils using 7% perchloric acid, and the histamine release was expressed as a percentage of the total. A response >16.5% of the spontaneous release was considered positive.

### Statistical Analysis

Data were analysed using SPSS software version 22.0 (IBM Corporation, New York, USA). Laboratory and clinical data of patients were compared using students t-test for numerical data and chi-square test and odds ratio test for categorical data. Correlation between variables was calculated using the Spearman’s correlation test.

The characteristics identified by the chi-square and student tests were used and combined to identify basophil test positive patients. For this analysis, we calculated the values of sensitivity, specificity, positive and negative predictive values for our sample.

Based on the data results, a decision tree was built using the CHAID algorithm (qui-squared automatic interaction detection) to enhance the ability to identify basophil test positive patients. The sensitivity, specificity, positive and negative predictive values were also calculated for these results.

In case of missing data, cases were excluded analysis by analysis, recalculating the N for the existing values. Results were reported as significant when the p was less than 0.05.

## Results

### The BAT, BHRA, or Both Are Positive in a Significant Subset of CSU Patients

Of the 85 patients with CSU (81% female, average age of 46 ± 16 years), 37 (44%) and 24 (28%) were positive in the basophil activation test (BAT) and the basophil histamine release assay (BHRA), respectively. Both tests showed a significant correlation (r=0.43, p<0.05), with a 70% match of the results (with 21% of all patients showing double positivity and 49% of all patients showing double negativity).

### CSU Patients With a Positive BAT or BHRA More Often Have Angioedema, Nocturnal Symptoms, and Wheals on Five or More Days per Week

Three clinical characteristics were statistically more frequent (p<0.05) in BAT-positive and in BHRA-positive compared to BAT/BHRA-negative patients (**Table 1**): 1) the occurrence of angioedema (BAT: 62% *vs* 35%; BHRA: 67% *vs* 39%); 2) the occurrence of nocturnal symptoms, sometimes causing premature awakening (BAT: 70% *vs* 50%; BHRA: 79% *vs* 51%); and 3) the presence of symptoms on at least five days per week (BAT: 92% *vs* 65%; BHRA: 92% *vs* 70%).

**Table 1:** Patients clinical and laboratorial data, according to the BAT and BHRA test result.

	BAT				BHRA			
	+ BAT (n=37)	- BAT (n=48)	Chi-square	Odds ratio	+ BHRA (n=24)	- BHRA (n=61)	Chi-square	Odds ratio
Positive ASST	23/37 (62%)	7/48 (15%)	**p<0.05**	9.622 (3.397-27.254)	13/24 (54%)	17/61 (28%)	**p<0.05**	3.059 (1.149-8.140)
Male Gender	7/37 (19%)	9/48 (19%)	p=0.984	1.011 (0.338-3.027)	4/24 (17%)	12/61 (20%)	p=0.750	0.817 (0.235-2.837)
Presence of angioedema	23/37 (62%)	17/48 (35%)	**p<0.05**	2.996 (1.231-7.292)	16/24 (67%)	24/61 (39%)	**p<0.05**	3.083 (1.143-8.315)
Presence of nocturnal CSU symptoms	26/37 (70%)	24/48 (50%)	**p<0.05**	2.364 (0.957-5.837)	19/24 (79%)	31/61 (51%)	**p<0.05**	3.677 (1.217-11.110)
Symptoms for ≥5 days/week	34/37 (92%)	31/48 (65%)	**p<0.05**	6.215 (1.660-23.274)	22/24 (92%)	43/61 (70%)	**p<0.05**	4.605 (0.979-21.664)
CSU for >6 months prior to the ASST	33/37 (89%)	44/48 (92%)	p=0.698	0.750 (0.175-3.222)	22/24 (92%)	55/61 (90%)	p=0.831	1.200 (0.225-6.406)
Hives duration >5 hours	18/37 (49%)	18/48 (38%)	p=0.302	1.579 (0.661-3.769)	13/24 (54%)	23/61 (37%)	p=0.167	1.953 (0.751-5.076)
Presence of anti-TPO/Tg autoantibodies	13/37 (35%)	9/47 (19%)	p=0.098	2.287 (0.848-6.165)	9/24 (38%)	13/60 (22%)	p=0.136	2.169 (0.775-6.074)
Presence of fT4/TSH abnormalities	7/37 (19%)	3/47 (6%)	p=0.078	3.422 (0.819-14.299)	3/24 (13%)	7/60 (12%)	p=0.915	1.082 (0.255-4.583)
Presence of ANA/anti-dsDNA autoantibodies	5/34 (15%)	4/47 (9%)	p=0.381	1.853 (0.459-7.490)	2/23 (9%)	7/58 (12%)	p=0.663	0.694 (0.133-3.619)
Presence of altered C3	2/33 (6%)	4/46 (9%)	p=0.663	0.677 (0.117-3.936)	3/23 (13%)	3/56 (5%)	p=0.208	2.842 (0.528-15.303)
Presence of altered C4	0/33 (0%)	2/46 (4%)	p=0.225	0.957 (0.899-1.017)	0/23 (0%)	2/56 (4%)	p=0.374	0.965 (0.918-1.014)
Presence of altered CH50	7/32 (22%)	9/45 (20%)	p=0.842	1.120 (0.368-3.404)	5/23 (22%)	11/54 (20%)	p=0.790	1.176 (0.356-3.891)
IgE<30 U/mL	15/37 (41%)	4/47 (9%)	**p<0.05**	7.330 (2.171-24.745)	9/24 (38%)	10/60 (17%)	**p<0.05**	3.000 (1.030-8.742)

BAT, basophil activation test; BHRA, basophil histamine release essay; ASST, autologous serum skin test; CSU, chronic spontaneous urticaria; anti-TPO, anti-thyroperoxidase; anti-Tg, anti-thyroglobulin; fT4, free thyroxine; TSH, thyroid stimulating hormone; ANA, antinuclear antibody; anti-dsDNA, anti-double stranded DNA.

Bold values highlight statistical significant values i.e. p < 0.05.

### CSU Patients With a Positive BAT or BHRA Have Higher Disease Activity and Impact, as Well as Higher Rates of Uncontrolled Disease

BAT-positive and BHRA-positive CSU patients had significantly higher disease activity as assessed by the UAS7 (p<0.05): 21.1 ± 9.7 *vs* 15.7 ± 10.6 for the BAT; and 22.2 ± 8.3 *vs* 16.4 ± 10.9 for the BHRA (**Table 2**). UAS7 values correlated (p<0.05), albeit weakly, with basophil activation in the BAT (r=0.130) and BHRA (r=0.230); the higher the disease activity, the greater the degree of basophil activation.

**Table 2:** Patients clinical and laboratorial data, according to the BAT and BHRA test result.

	BAT			BHRA		
	+ BAT (n=37)	- BAT (n=48)	Student test	+ BHRA (n=24)	- BHRA (n=61)	Student test
Age (years)	44 ± 15	47 ± 17	p=0.332	45 ± 16	46 ± 16	p=0.977
UAS7 (mean ± SD)	21.1 ± 9.7	15.7 ± 10.6	**p<0.05**	22.2 ± 8.3	16.4 ± 10.9	**p<0.05**
DLQI (mean ± SD)	9.3 ± 6.8	6.5 ± 5.6	**p<0.05**	10.3 ± 6.1	6.7 ± 6.1	**p<0.05**
UCT (mean ± SD)	7.8 ± 4.1	9.3 ± 3.9	**p<0.05**	7.7 ± 3.9	9.0 ± 4.1	**p<0.05**
Total serum IgE (U/mL)	91 ± 91	395 ± 961	**p<0.05**	74 ± 69	335 ± 858	**p<0.05**
	+ BAT (n=37)	- BAT (n=47)	Student test	+ BHRA (n=24)	- BHRA (n=61)	Student test
Ratio IgG-anti-TPO/Total IgE	5.7 ± 16.4	0.2 ± 1.0	**p<0.05**	6.2 ± 17.1	1.2 ± 7.1	p=0.192

BAT, basophil activation test; BHRA, basophil histamine release essay; UAS7, urticaria activity score 7; DLQI, dermatological life quality index; UCT, urticaria control test.

Bold values highlight statistical significant values i.e. p < 0.05.

Disease impact, i.e. quality of life impairment assessed with the DLQI, was higher in CSU patients with a positive BAT or BHRA (p<0.05): 9.3 ± 6.8 *vs* 6.5 ± 5.6 for the BAT; and 10.3 ± 6.1 *vs* 6.7 ± 6.1 for the BHRA. CSU patients with a positive BAT or BHRA showed lower levels of disease control, as assessed by the UCT (p<0.05): 7.8 ± 4.1 *vs* 9.3 ± 3.9 for the BAT, and 7.7 ± 3.9 *vs* 9.0 ± 4.1 for the BHRA.

### CSU Patients With a Positive BAT/BHRA Have Lower Levels of Total Serum IgE and Higher Rates of Thyroid Autoantibodies

BAT-positive and BHRA-positive CSU patients had significantly lower levels of total serum IgE as compared to negative patients (p<0.05): 91 ± 91 *vs* 395 ± 961 U/mL and 74 ± 69 *vs* 335 ± 858 U/ml, respectively (**Table 2**). In fact, the rate of CSU patients with a total serum IgE below 30 U/mL was significantly higher in BAT-positive and BHRA-positive patients (p<0.05): 41% *vs* 9% and 38% *vs* 17%, respectively (**Table 1**).

The rates of patients with autoantibodies to thyroid peroxidase (IgG-anti-TPO) or to thyroglobulin (IgG-anti-Tg) were higher in BAT-positive or BHRA-positive CSU patients, although this was not statistically significant: 35% *vs* 19% for the BAT, and 38% *vs* 22% for BHRA (**Table 1**). Also, the ratio of IgG-anti-TPO to total serum IgE was higher in BAT or BHRA positive patients: 5.7 ± 16.4 *vs* 0.2 ± 1.0 for the BAT (p<0.05), and 6.2 ± 17.1 *vs* 1.2 ± 7.1 for the BHRA (p=0.192; **Table 2**).

### Autologous Serum Skin Testing Does Not Improve Patient Profiling by Basophil Testing

Basophil test-positive patients who also tested positive in the autologous serum skin test (ASST), when compared to those who were negative for both, showed a similar profile to the comparison between patients who were basophil test-positive and basophil test-negative, regardless of their ASST result, i.e. higher disease activity (UAS7 22.3 ± 9.5 *vs* 15.0 ± 10.3, p<0.05), higher disease impact (DLQI 9.7 ± 7.6 *vs* 6.2 ± 5.6, p<0.05), lower disease control (UCT 7.3 ± 4.2 *vs* 9.8 ± 3.4, p<0.05), lower total serum IgE (85.6 ± 97.8 *vs* 455.1 ± 1090.5, p<0.05), and higher ratio of IgG-anti-TPO to total serum IgE (3.0 ± 6.7 *vs* 0.3 ± 1.1, p<0.05). The combined use of basophil and autologous serum skin testing identified the same features in double positive patients, as compared to those linked to positive basophil testing only (**Table 3**).

**Table 3:** Positive predictive value, negative predictive value, sensitivity, and specificity of a positive basophil test (BAT or BHRA) according to patients’ characteristics.

Patients’ characteristics	Positive predictive value	Negative predictive value	Sensitivity	Specificity
ASST(+)	80.0	61.8	53.3	85.0
UAS7≥16	59.6	55.3	62.2	52.5
Angioedema	62.5	55.6	55.6	62.5
Nocturnal symptoms	62.0	60.0	68.9	52.5
>5 days/week	60.6	73.7	88.9	35.0
Anti-TG/TPO	59.1	48.4	28.9	76.9
TSIgE<30	78.9	53.8	33.3	89.7
TSIgE<30 + Anti-TG/TPO	100.0	52.0	20.0	100
ASST(+) + Angioedema	86.4	58.7	42.2	92.5
ASST(+) + Anti-TG/TPO	90.0	50.7	20.0	97.4
ASST(+) + TSIgE<30	100.0	54.2	26.7	100.0

ASST, autologous serum skin test; anti-TPO, anti-thyroperoxidase; anti-Tg, anti-thyroglobulin; UAS7, urticaria activity score 7; TSIgE, Total serum IgE in (U/mL).

### The ASST, by Itself, Is Not a Good Predictor of a Positive Basophil Test

Of the 85 CSU patients we investigated, 30 (35%) had a positive autologous skin test (ASST+). The ASST showed a weak correlation with the BAT (r=0.37, p<0.05) and with the BHRA (r=0.29, p<0.05). The probability (sensitivity) for a positive ASST to identify a patient with a positive BAT or BHRA was 62% and 56%, respectively.

### The ASST, Combined With Other Clinical and Routine Laboratory Markers, Can Help to Select Patients for Basophil Testing

Next, we tested if the clinical features and laboratory markers linked to a positive BAT or BHRA predicted which patients would have a positive basophil test. By themselves, the sensitivity and specificity of angioedema, nocturnal wheals, wheals on five or more days per week, low IgE, elevated thyroid autoantibodies and high disease activity ranged from 29% to 89% and 35% to 90% for predicting a positive BAT or BHRA, respectively (**Table 4**).

**Table 4:** Patients clinical and laboratorial data, according to ASST plus basophil test double-positivity or double-negativity.

	ASST and basophil test double-positive patients (n=24)	ASST and basophil test double-negative patients (n=36)	Chi-square	Odds ratio	Student test
Male Gender	3 (13%)	7 (19%)	p=0.480	0.592 (0.137-2.560)	–
Presence of angioedema	19 (79%)	12 (33%)	**p<0.05**	7.600 (2.279-25.345)	–
Presence of nocturnal CSU symptoms	17 (71%)	16 (44%)	**p<0.05**	3.036 (1.012-9.107)	–
Symptoms for ≥5 days/week	23 (96%)	23 (64%)	**p<0.05**	13.000 (1.569-107.708)	–
Hives duration >5 hours	16 (67%)	13 (36%)	**p<0.05**	3.538 (1.193-10.499)	–
Presence of anti-TPO/Tg autoantibodies	9 (38%)	10 (28%)	p=0.471	1.500 (0.497-4.528)	–
Presence of fT4/TSH abnormalities	5 (21%)	3 (8%)	p=0.177	2.807 (0.602-13.091)	–
Presence of ANA/anti-dsDNA autoantibodies	2 (8%)	4 (11%)	p=0.780	0.775 (0.130-4.633)	–
IgE<30 U/mL	12 (50%)	4 (11%)	**p<0.05**	7.750 (2.084-28.815)	–
UAS7 (mean ± SD)	22.3 ± 9.5	15.0 ± 10.3	–	–	**p<0.05**
DLQI (mean ± SD)	9.7 ± 7.6	6.2 ± 5.6	–	–	**p<0.05**
UCT (mean ± SD)	7.3 ± 4.2	9.8 ± 3.4	–	–	**p<0.05**
Total serum IgE (U/mL)	85.6 ± 97.8	455.1 ± 1090.5	–	–	**p<0.05**
Ratio IgG-anti-TPO/Total IgE	3.0 ± 6.7	0.3 ± 1.1	–	–	**p<0.05**

ASST, autologous serum skin test; CSU, chronic spontaneous urticaria; anti-TPO, anti-thyroperoxidase; anti-Tg, anti-thyroglobulin; fT4, free thyroxine; TSH, thyroid stimulating hormone; ANA, antinuclear antibody; anti-dsDNA, anti-double stranded DNA; UAS7, urticaria activity score 7; DLQI, dermatological life quality index; UCT, urticaria control test.

Bold values highlight statistical significant values i.e. p < 0.05.

When the ASST was combined with angioedema, elevated thyroid autoantibodies or low IgE, it predicted a positive basophil test (either BAT or BHRA) with 92.5%, 97.4% and 100% specificity, respectively. In addition, patients with low IgE and elevated thyroid autoantibodies also had 100% specificity of identifying a positive basophil test. These combinations, however, showed low sensitivities, excluding many basophil test-positive patients.

**Figure 1:**
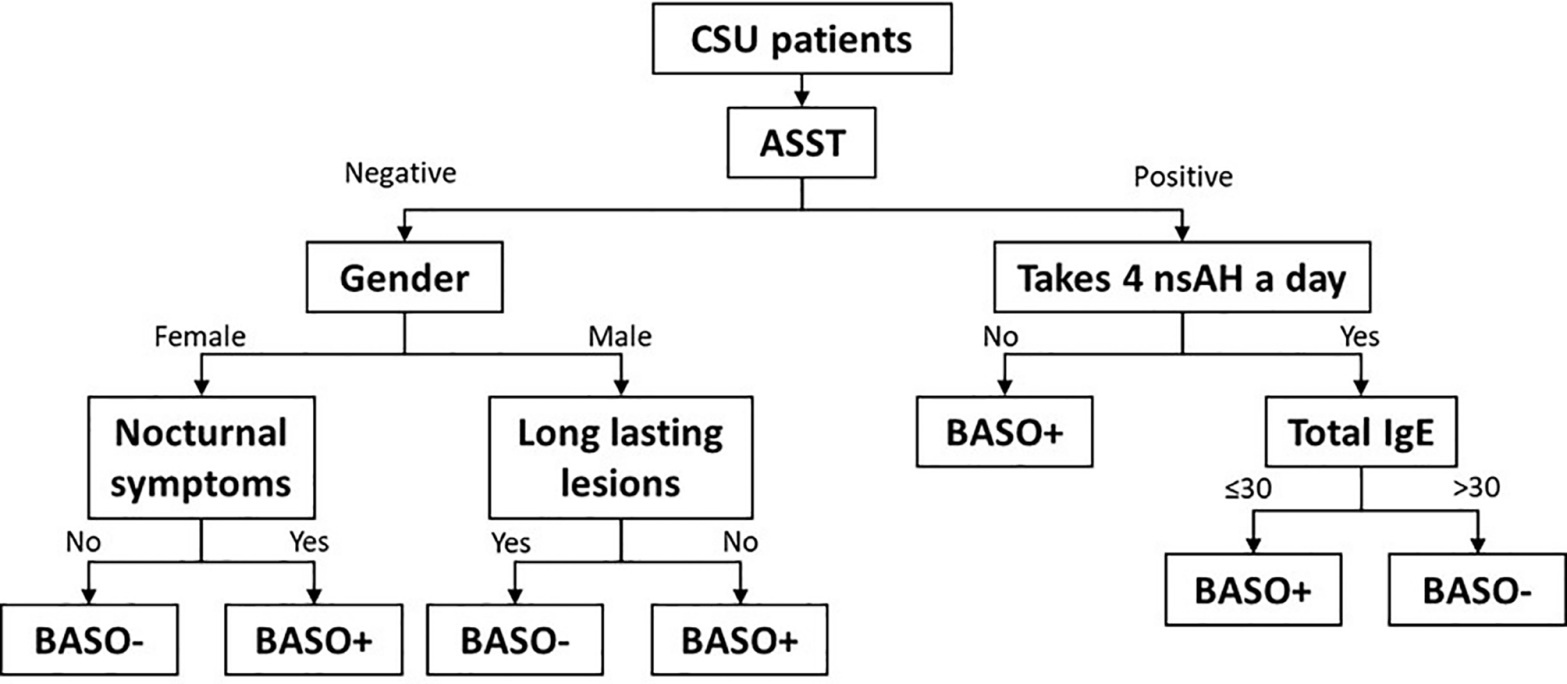
Decision tree assessing patients with the highest probability of being basophil-positive (BASO+) and basophil-negative (BASO-) patients. BASO+ were accurately predicted by this model with a sensitivity of 76.7% (IC95% 61.4-88.2), a specificity of 64.3% (IC95% 48.0-78.5), a positive predictive value of 68.8% (IC95% 58.7-77.3), and a negative predictive value of 73.0% (IC95% 60.0-82.9).

To optimize the identification of patients with a positive basophil test (either BAT or BHRA), a decision tree was built using the CHAID algorithm (qui-squared automatic interaction detection). Applying the decision tree, the CSU patients were sequentially divided according to their clinical characteristics, until a final branch, after which patients were labelled as having either a positive basophil test or a negative basophil test (**Figure 1**).

Comparing the patients who were identified by this decision tree as being basophil test positive or negative with the actual BAT and BHRA results, we calculated that this decision tree showed a sensitivity of 76.7% and a specificity of 64.3% for correctly identifying the basophil test result of the patients.

## Discussion

The results of the CORSA study show that basophil test-positive patients (either BAT or BHRA) represent a distinct subset of CSU patients, with characteristics of TIIbaiCSU detailed by the recent PURIST study (4).

In our cohort, 44% and 28% of the patients were BAT-positive and BHRA-positive, respectively. This this is expected, as most studies report basophil test-positive results in 25% to 45% of the cases (8, 9, 23–25), with some studies going as high as 64% (26). However, even though our BAT and BHRA tests showed significant correlation and a 70% match in the results, this correlation was not perfect. The precise reason for this mismatch is currently unknown, however, not unexpected.

There are few studies directly comparing the two basophil tests in CSU. A previous study by our Department evaluated the correlation between ASST and BAT positivity in 48 CSU patients, and found a correlation coefficient of 0.79 (27). A study by Altrich et al. calculated a correlation coefficient between both tests of r=0.54, even though the concordance (positive versus negative) was high (75%); which is similar to the results presented in this study (28). Another study, by Yasnowsky and co-workers, showed a correlation of r=0.6, although in this case the BAT test was evaluated using CD203c expression, which may have influenced these results (29). Szegedi and colleagues published results with a higher degree of correlation: r=0.91 when an atopic donor was used and r=0.7 when a non-atopic donor was used (30). However, these results highlight an important caveat; the correlation between basophil test results can change significantly with the characteristics of the donor of the basophils used. Additionally, in Szegedi’s study, the same atopic and non-atopic donor were used for all the tests. In our study, different donors were used for the BAT and the BHRA, which can further explain the correlation discrepancies.

Not only do the BAT and BHRA use different techniques, which can impact the degree of basophil activation, it is hard to quantify the impact of using different basophil donors. This is also true for the tests individually. In the BAT and BHRA, the same serum tested at the same time with different donors, produces different degrees of basophil activation. The basophil test (BAT and BHRA) and ASST mismatch, on the other hand, was expected and has been described in the literature (24, 28–32).

The recent PURIST study, the first study to identify TIIbaiCSU patients using all three defining tests, provided important evidence of the distinctive characteristics of these patients. It showed that this patient subgroup presented higher disease activity (i.e. UAS7), lower total serum IgE, higher rates of thyroid autoantibodies and a higher IgG-anti-TPO to total serum IgE ratio (4). The results of the CORSA study show that our basophil test-positive patients exhibit a phenotype that is distinct from that of our basophil test-negative patients, and which is in agreement with the TIIbaiCSU profile of the patients of the PURIST study. Our basophil test-positive patients presented higher disease activity (UAS7), higher disease impact (DLQI), lower disease control (UCT), lower total serum IgE, higher rates of thyroid autoantibodies and a higher IgG-anti-TPO to total serum IgE ratio.

However, these results were based solely on basophil testing, one of the three tests required to identify TIIbaiCSU. Therefore, we re-analysed the phenotypes after adding the ASST (a second criterion for TIIbaiCSU). The results remained unchanged. This suggests that the basophil test is the more significant test for identifying patients with this more severe phenotype. In fact, most previous studies, which reported higher disease severity (24, 25), lower disease control (11), elevated thyroid autoantibodies (9, 14, 28), a non-response or slower response to omalizumab, and a better response to cyclosporin (8–11), based their findings on a single basophil test (BAT or BHRA) and not on all three tests needed to identify TIIbaiCSU. This makes the case for why basophil testing is so important for evaluating CSU patients for type IIb autoimmunity and how it can help to identify cases of severe CSU early on.

Using both the BAT and the BHRA, we were also able to identify novel clinical characteristics of basophil test-positive patients. The CORSA study basophil test-positive patients showed a higher frequency of angioedema, nocturnal symptoms, and wheals on five or more days per week; characteristics which may have influenced the poorer results on the UAS7, DLQI and UCT scores.

Despite its relevance, basophil testing meets real challenges as a solution for widespread implementation for all CSU patients, namely in terms of its availability, cost, and the high prevalence of CSU in the population. Identifying markers of basophil test-positive patients would be of great use in clinical practice, as they could select the best group of patients in whom to perform the test.

The ASST, the first attempt at such a marker, proved insufficient (1, 4). Our results confirm this and show a sensitivity of only 53% at identifying BAT+ and/or BHRA+ patients. In an attempt to improve these results, we used combinations of the ASST with the characteristics we identified in our basophil test-positive patients. ASST-positive patients with simultaneous presence of thyroid autoantibodies or a total serum IgE<30, showed a ≈100% probability (specificity) of having a positive basophil test. The combination of total serum IgE<30 and the presence of thyroid autoantibodies also showed 100% specificity. However, these combinations showed a very low sensitivity, and would leave out more basophil test-positive patients than the ASST.

A stepwise approach, employing a decision tree, using the characteristics associated with basophil test positivity, correctly identified most of our patients: it correctly identified 33 of 48 basophil test-positive patients and 27 of 37 basophil test-negative patients. This decision tree is easy to use and easily replicable and can be a promising tool to many physicians treating CSU patients. By this model, restricting the patients tested with a basophil test to those indicated, a physician could: 1) significantly reduce the number of patients in whom to ask for a basophil test to a cost-effective number, and 2) increase the number of positive basophil tests from the 25-45% average described in the literature for the whole CSU population, to about 70%.

This study has some limitations. It has a limited number of patients, from a single center, and these findings were not tested in a control group. There is a need for further studies to determine why TIIbaiCSU or a basophil-positive patient presents with more severe CSU, lower IgE, and higher thyroid autoantibodies. In addition, these findings need to be corroborated by future studies in larger and more diverse patient populations, and the proposed decision tree should be looked at as an initial attempt to identify basophil-positive patients, to be perfected by future studies. The identification of additional easily available clinical/laboratory parameters and their insertion in the model might be useful to increase the specificity and sensitivity of this algorithm.

In conclusion, basophil testing identifies a distinct subset of CSU patients, consistent with TIIbaiCSU. Using clinical and laboratory characteristics of TIIbaiCSU, it is possible to identify patients who are likely to have a positive basophil test, allowing for a more patient-specific approach to CSU patients in the future and a more cost-effective use of the basophil tests.

## Data Availability Statement

The datasets presented in this article are not readily available because it requires authorization by ethics committee. Requests to access the datasets should be directed to corresponding author JM.

## Ethics Statement

The studies involving human participants were reviewed and approved by Comissão de Ética do Centro Hospitalar Universitário Lisboa Norte e Centro Académico Médico de Lisboa (Ethics Committee authorization references 129/17 and 339/19). The patients/participants provided their written informed consent to participate in this study.

## Author Contributions

JM, KB, PS, and MM contributed to conception and design of the study. JM, PS, and KB organized the database, performed the statistical analysis. JM and MM wrote the first draft of the manuscript. KB, PS, MS, IW, JS, SA, AW, CC, and MP-B wrote sections of the manuscript. All authors contributed to the article and approved the submitted version.

## Funding

All of the costs for this study were supported by our institutions, without external funding.

## Conflict of Interest

KB is employed by RefLab ApS. PS is acting as scientific advisor for RefLab and EP Medical. MM is or recently was a speaker and/or advisor for and/or has received research funding from Allakos, Aralez, AstraZeneca, CSL Behring, FAES, Genentech, Menarini, Novartis, Leo Pharma, Lilly, Moxie, MSD, Roche, Sanofi, UCB, and Uriach. SA is or recently was a speaker and/or advisor for and/or has received research funding from Allakos, AstraZeneca, CSL Behring, Novartis and Moxie.

The remaining authors declare that the research was conducted in the absence of any commercial or financial relationships that could be construed as a potential conflict of interest.

The handling Editor declared a past co-authorship with one of the authors MM.

## Publisher’s Note

All claims expressed in this article are solely those of the authors and do not necessarily represent those of their affiliated organizations, or those of the publisher, the editors and the reviewers. Any product that may be evaluated in this article, or claim that may be made by its manufacturer, is not guaranteed or endorsed by the publisher.

## Abbreviations

CSU, Chronic spontaneous urticaria; BAT, basophil activation test; BHRA, basophil histamine release assay; ASST, autologous serum skin test; UAS 7, urticaria activity score 7; DLQI, dermatology life quality index; UCT, urticaria control test; anti-TPO, anti-thyroperoxidase autoantibody; anti-Tg, anti-thyroglobulin autoantibody; TIaiCSU, type I autoimmune CSU; TIIbaiCSU, type IIb autoimmune CSU; ANA, antinuclear antibody; anti-ds-DNA, anti-double stranded DNA antibody; TsIgE, total serum IgE.
